# Measures of Psychological Mindedness: A Systematic Review of Psychometric Properties

**DOI:** 10.1002/cpp.70064

**Published:** 2025-03-23

**Authors:** Joshua Eli Thompson, Gillian Haddock, Katherine Berry

**Affiliations:** ^1^ Division of Psychology and Mental Health School of Health Sciences, Faculty of Biology, Medicine and Health, Manchester Academic Health Science Centre, University of Manchester Manchester UK; ^2^ Pennine Care NHS Foundation Trust Ashton‐Under‐Lyne UK; ^3^ Greater Manchester NHS Foundation Trust, Research and Innovation, Rawnsley Building, Manchester Royal Infirmary Manchester UK

**Keywords:** measures, psychological mindedness, psychometric, reliability, review, validity

## Abstract

**Objective:**

Psychological mindedness has been positively associated with psychological wellbeing and positive outcomes in psychological therapy. Valid and reliable measures of psychological mindedness are needed for accurate measurement of the construct. This paper is the first to provide a comprehensive review of existing measures of psychological mindedness.

**Methods:**

The review protocol was pre‐registered and systematic with methods reported according to PRISMA criteria. The quality of studies reporting on psychometric properties of measurement tools was evaluated against the COSMIN criteria.

**Results:**

Twenty‐three studies relating to six measures of psychological mindedness were included in the review. No measure demonstrated sufficient evidence when evaluated against all COSMIN measurement criteria. However, the Balanced Index of Psychological Mindedness (BIPM) demonstrated the most robust psychometric properties with sufficient evidence of structural validity and internal consistency demonstrated through studies of high quality.

**Conclusions:**

Whilst the BIPM demonstrated the most robust measurement properties, further research is needed in relation to its content validity, cross‐cultural validity, and responsiveness. The BIPM also does not incorporate ‘other‐oriented’ psychological mindedness. Alternative measures such as the PMS and PMAP are available to measure psychological mindedness towards others but have less sufficient evidence of psychometric rigour.

**Summary**

- Psychological mindedness has been defined and operationalised as both ‘self‐oriented’ and ‘other‐oriented’ within measures.
- The BIPM was identified as having the strongest psychometric rigour of measures evaluated in this review, with sufficient high‐quality evidence of structural validity and internal consistency.
- The BIPM is largely ‘self‐oriented’ and was the only measure of psychological mindedness included in the review that does not incorporate an ‘other’ oriented element to the measure.
- Evaluation of construct validity of measures included within this review indicated a positive association between psychological mindedness and mental wellbeing.

## 1 Introduction

Psychological mindedness has been defined as ‘the ability to see relationships amongst thoughts, feelings, and actions, with the goal of learning the meanings and causes of experiences and behaviours’ (Appelbaum 1973, 36). Psychological mindedness has been described as an ‘explicit’ process in which a person is actively engaged in understanding psychological experiences (Choi‐Kain and Gunderson 2008). The concept is derived from psychodynamic theories and has long been considered by therapists as a desirable trait in clients, even prior to the development of measures relating to psychological mindedness and despite ambiguity in its definition (Lower et al. 1972). Psychological mindedness has been positively associated with mental wellbeing (Trudeau and Reich 1995) and has been reported as a protective factor against negative effects in therapy such as worsening of symptoms (Pourová et al. 2023). A higher level of psychological mindedness of individuals attending for psychological therapy has been associated with reductions in depression (Kishon et al. 2019) and anxiety (Kosasih et al. 2023).

Psychological mindedness has been considered alongside other concepts, including mentalization (Choi‐Kain and Gunderson 2008), cognitive and emotional empathy (Beitel et al. 2005), insight (Grant 2001; Nyklicek and Denollet 2009), alexithymia (Shill and Lumley 2002; Taylor et al. 1989), mindfulness (Beitel et al. 2005) and private self‐consciousness (Grant 2001). Despite overlap, psychological mindedness has been considered a distinct concept from these constructs. Where mentalization involves both implicit (unconscious and automatic) and explicit (conscious and deliberate) processes (Choi‐Kain and Gunderson 2008) and does not incorporate a person's interest in reflection, psychological mindedness emphasises a person's interest and motivation to engage in psychological thinking (Choi‐Kain and Gunderson 2008; Conte et al. 1990; Nyklicek and Denollet 2009). Where insight may be the result of psychological reflection and is incorporated within the concept of psychological mindedness, psychological mindedness can be seen as both the result of and the process of developing insight (Appelbaum 1973; Nyklicek and Denollet 2009). Where empathy refers to understanding a person's emotional or cognitive experience, psychological mindedness is the consideration of factors which led to said experience (Beitel et al. 2005). Unlike alexithymia which is characterised as an inability to recognise and describe emotions, psychological mindedness has been described as the interest and ability to understand behaviour, thoughts and emotions (Kishon et al. 2019; Nyklicek and Denollet 2009). Both psychological mindedness and private self‐consciousness involve examination of one's mental and emotional processes. However, Grant (2001) identified a distinction in which psychological mindedness is directed at explanation or understanding of one's own and others' behaviour, whereas private self‐consciousness involves awareness of one's own thoughts, feelings and behaviour. Grant (2001) proposed that private self‐consciousness is one of several constructs that combine to form psychological mindedness.

Although it is possible to distinguish psychological mindedness from the other related concepts, conceptual questions remain about the construct of psychological mindedness. For example, a person's level of psychological mindedness has generally been considered as a static trait (Appelbaum 1973). However, research indicates that a person's level of psychological mindedness can change following psychological intervention (Nyklicek et al. 2010). In addition, Appelbaum's original definition refers to psychological mindedness as the ability to see the relationship between thoughts, feelings and actions of one's own behaviour and experiences (Appelbaum 1973). However, other definitions of psychological mindedness have since incorporated an ‘other’ oriented aspect of the concept with definitions including reflection upon the meaning and motivation of behaviour, thoughts and feelings of oneself and others (Conte et al. 1990; Farber 1985), and the ability to identify dynamic components and to connect them to one's own and other's difficulties (McCallum and Piper 1990).

Various methods have been used to measure psychological mindedness in individuals including self‐report and observer‐rated methods. These measures have been summarised elsewhere in the literature in a small number of relatively outdated conceptual overviews (Conte and Ratto 1997; Rai et al. 2015). The reviews indicated the need for further research around measures of psychological mindedness and for further standardisation of the concept of psychological mindedness (Conte and Ratto 1997; Rai et al. 2015). However, these overviews did not incorporate a systematic review of studies reporting evidence of the psychometric properties of measures of psychological mindedness, nor did they appear to use a quality appraisal tool to assess the methodological quality of studies. The absence of a systematic review of measures of psychological mindedness incorporating a quality appraisal of studies means there is a lack of clarity in the literature regarding the quality of evidence reporting on the psychometric properties of measures of psychological mindedness.

For psychological mindedness to be considered as a potential mechanism or outcome of psychotherapy it is important that instruments measuring psychological mindedness are valid and reliable. A systematic review regarding the quality of studies assessing the validity and reliability of known measures of psychological mindedness would support appropriate use of psychological mindedness measures in research and clinical practice and would help to identify future avenues of research regarding the concept and measurement of psychological mindedness. This systematic review therefore aims to identify current methods of assessing psychological mindedness and to assess the reliability and validity of the tools available. For the purpose of the review, we defined psychological mindedness as a person's capacity, inclination and motivation to understand theirs and others' thoughts and feelings in relation to their experiences and behaviour. This definition draws upon a number of other definitions of psychological mindedness in the literature. It emphasises Appelbaum's (1973) definition of understanding ‘thoughts, feelings and actions’ with the ‘goal of understanding the meaning and causes of experience and behaviour’. It incorporates Nyklicek and Denollet's (2009) conceptualization of psychological mindedness as ‘insight’ and ‘interest’ in understanding experiences. It draws on Farber's (1985) interpretation of psychological mindedness as a ‘disposition’ to reflect upon the meaning of thoughts, feelings and behaviour of oneself and others. Our definition also reflects Nyklicek et al.'s (2010) recognition of the potential for change in a person's level of psychological mindedness.

### 1.1 Objectives

This systematic review aimed to

- identify the measures available that aim to assess psychological mindedness in individuals,
- to use a standardised assessment tool to evaluate the methodological quality of studies investigating the psychometric properties of a measure of psychological mindedness,
- to evaluate the overall quality of evidence relating to measures of psychological mindedness,
- to summarise the results and make recommendations for the use of measures of psychological mindedness based on the evaluation of measurement properties and key issues relating to the conceptual understanding of psychological mindedness and
- to make recommendations for future research in the measurement of psychological mindedness.

## 2 Method

The review protocol was pre‐registered with PROSPERO (CRD42024510809). The methods were reported in accordance with PRISMA guidelines (Page et al. 2021) and followed COSMIN guidelines for systematic reviews of patient‐reported outcome measures (PROMs; Prinsen et al. 2018). The COSMIN guidelines were designed for evaluating PROMs but can be used flexibly to evaluate the psychometric properties of other forms of measures such as clinician or researcher‐administered measures (Mokkink, de Vet, et al. 2018). The approach has the advantage of offering a structured approach to evaluating the psychometric properties of measures and for assessing the quality of studies reporting on psychometric properties of measures (Prinsen et al. 2018).

### 2.1 Eligibility Criteria

Inclusion criteria were (1) article is written in English language, (2) article is published in a peer review journal and (3) article reports on psychometric properties (reliability and validity) of a self‐report or clinician or researcher‐administered measure of psychological mindedness including translated versions of psychological mindedness measures.

Exclusion criteria were (1) review papers, case studies, book chapters, monographs, dissertations, or conference extracts; (2) studies which solely use the measure to validate another instrument; and (3) measures which were not developed to distinctly assess psychological mindedness, such as measures of concepts with conceptual overlap with psychological mindedness, but for which there is agreement in the literature that they remain distinct concepts (e.g., mentalization, empathy and private self‐consciousness).

### 2.2 Search Strategy

PsycInfo, Web of Science, Embase, CINAHL, MEDLINE and Scopus databases were originally searched in September 2023. Searches were repeated in September 2024 with no additional papers identified for inclusion in the review. Language limits were not applied in searches and the authors sought to identify English versions of articles not written in English language. Search terms were applied to ‘full text’ across all databases. Full text searches were applied as the authors were aware of potentially relevant papers that did not specifically report psychological mindedness terms in titles, key words and abstracts and thus did not appear in searches limited to these fields. Search terms were developed in line with COSMIN guidelines which identifies key elements, including (1) construct, (2) type of instrument and (3) measurement properties. The search terms did not incorporate the fourth ‘population’ element of the COSMIN guidelines as the authors aimed to evaluate the psychometric properties of measures of psychological mindedness in all populations. The reference lists and citations of eligible studies were also reviewed.

### 2.3 Selection Process

The first author screened titles and abstracts of all papers returned via searches and articles were selected for a review of full texts based on the eligibility criteria. An independent researcher (doctorate‐level clinical psychology trainee) screened 10% of all titles and abstracts returned via searches (*n* = 233). Ninety‐eight percent agreement was achieved in identifying full texts to be reviewed (*k* = 0.938). Inconsistencies were discussed and reviewed against the eligibility criteria until agreement was reached. All full text articles were reviewed by the research team against the eligibility criteria.

### 2.4 Data Extraction

Data extraction was informed by the COSMIN taxonomy of measurement properties (Table 1) which identifies psychometric properties across three domains of reliability, validity and responsiveness (Prinsen et al. 2018).

**TABLE 1:** COSMIN definitions of domains and measurement properties (Mokkink, Prinsen, et al. 2018).

Domain	Measurement property	Aspect of a measurement property	Definition
**Reliability**			The degree to which the measurement is free from measurement error
	Internal consistency		The degree of the interrelatedness among the items
	Reliability		The proportion of the total variance in the measurements which is due to ‘true’ differences between patients
	Measurement error		The systematic and random error of a patient's score that is not attributed to true changes in the construct to be measured
**Validity**			The degree to which a Patient‐reported Outcome Measure (PROM) measures the construct(s) it purports to measure
	Content validity		The degree to which the content of a PROM is an adequate reflection of the construct to be measured
		Face validity	The degree to which (the items of) a PROM indeed looks as though they are an adequate reflection of the construct to be measured
	Construct validity		The degree to which the scores of a PROM are consistent with hypotheses *(for instance, with regard to internal relationships, relationships to scores of other instruments or differences between relevant groups)* based on the assumption that the PROM validly measures the construct to be measured
		Structural validity	The degree to which the scores of a PROM are an adequate reflection of the dimensionality of the construct to be measured
		Hypothesis testing	See construct validity
		Cross‐cultural validity	The degree to which the performance of the items on a translated or culturally adapted PROM are an adequate reflection of the performance of the items of the original version of the PROM
	Criterion validity		The degree to which the scores of a PROM are an adequate reflection of a ‘gold standard’
**Responsiveness**			The ability of a PROM to detect change over time in the construct to be measured
	Responsiveness		Idem responsiveness
Interpretability*			Interpretability is the degree to which one can assign qualitative meaning—that is, clinical or commonly understood connotations—to a PROM's quantitative scores or change in scores.

* Interpretability is not considered a measurement property, but an important characteristic of a measurement instrument.

### 2.5 Assessment of Measurement Properties

**FIGURE 1:**
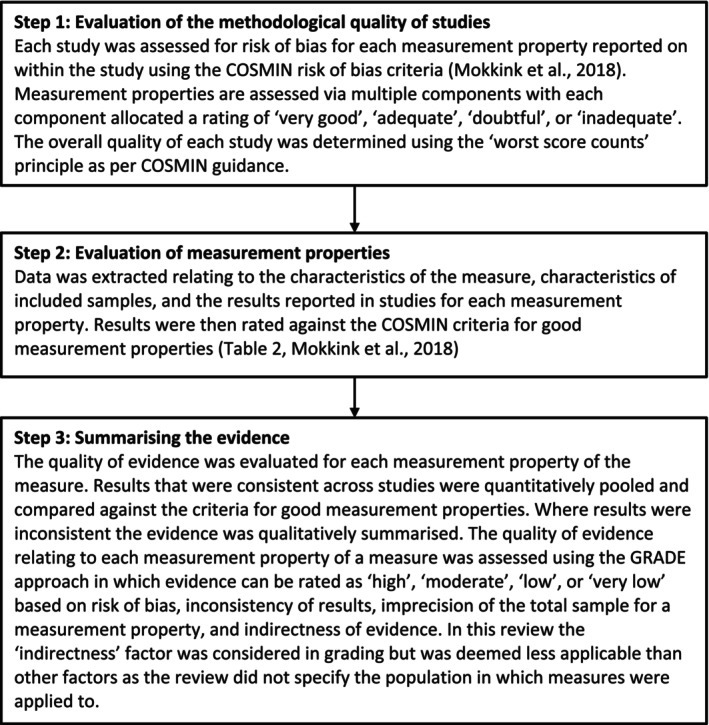
COSMIN methodology for the evaluation of measurement properties (Mokkink, Prinsen, et al. 2018).

The COSMIN methodology (Mokkink, Prinsen, et al. 2018) describes the assessment of measurement properties in three steps which the author followed. These steps are presented in Figure 1.

For step one (evaluation of the methodological quality of studies using the COSMIN risk of bias checklist), the quality of each study was rated as ‘very good’, ‘adequate’, ‘doubtful’ or ‘inadequate’. This rating was based on various criteria as outlined in the COSMIN guidelines (Mokkink, de Vet, et al. 2018; Prinsen et al. 2018; Terwee et al. 2018). For example, the rating of a study reporting on the reliability of a measure was assessed in consideration of factors relating to the study design such as stability of the study population during the study period, appropriateness of the time interval between administering the measure in the study, and consistency in test conditions. The quality rating also considered factors relating to the statistical methods reported in the study such as whether an intraclass correlation coefficient or Cohen's kappa was calculated and, if relevant, whether a weighted kappa was calculated and adequately described. In this instance, if a study provided evidence that the study population was stable throughout the study (very good), evidence that the time interval was appropriate (very good), but a lack of evidence that the test conditions were stable (doubtful), the study would receive a rating of ‘doubtful’ based on the ‘lowest score counts’ rating process.

Similarly, for a study reporting on the construct validity of a measure, the quality of the study was also rated in consideration of design requirements and statistical methods. Design requirements considered for studies reporting on construct validity included the clarity of the construct measured by the comparator instrument and the quality of evidence relating to the measurement properties of the comparator instrument. Consideration of the statistical methods used in a study reporting on construct validity related to whether the statistical method was appropriate and adequate for the hypothesis to be tested. For example, if a study used a comparator measure which clearly reflected the comparator concept (very good), with a strong evidence base for sufficiency of the comparator measure's psychometric properties (very good), and an appropriate statistical method was used to test the study hypotheses (very good), and no evidence of other important flaws in the design or statistical analysis (very good), the study would receive a rating of ‘very good’. Details of rating requirements for all COSMIN measurement properties are available in COSMIN guidelines (Mokkink, de Vet, et al. 2018; Prinsen et al. 2018; Terwee et al. 2018).

See Table 2 for the COSMIN criteria for good measurement properties (Mokkink, Prinsen, et al. 2018) used in step 2 (evaluation of measurement properties). The three‐step process was repeated for each measurement property of each measure included in the review. However, in line with recommended modifications for using the COSMIN guidance with measures such as observer‐rated or clinician‐reported measures, some aspects were modified when assessing the quality of evidence for clinician and observer‐rated measures. Specifically, factors relating to the inter‐rater reliability of clinician and researcher‐administered measures were considered under the ‘any other flaws in the design or statistical methods’ item of the quality appraisal for studies reporting on reliability. The COSMIN guidance identifies criterion validity as a measurement property to be evaluated. However, the guidance indicates that a ‘gold standard’ measure of a concept is required for other measures to be evaluated against (Mokkink, de Vet, et al. 2018). The authors felt unable to identify a ‘gold standard’ measure of psychological mindedness due to variance in the literature in how psychological mindedness has been operationalised and a lack of previous reviews appraising the quality of evidence reporting on the psychometric properties of measures. Therefore, in accordance with COSMIN guidelines, studies comparing two measures of psychological mindedness were evaluated using the ‘hypothesis testing for construct validity’ criteria rather than criteria for ‘criterion validity’.

In line with the COSMIN guidance, measurement properties were evaluated in the following order: measure development, content validity, structural validity, internal consistency, cross‐cultural validity/measurement invariance, reliability, measurement error, criterion validity, hypothesis testing for construct validity and responsiveness. It was noted that measure development is not a measurement property but should be considered when evaluating content validity (Mokkink, de Vet, et al. 2018).

Relating to hypothesis testing for construct validity, the authors reviewed the evidence base relating to the relationship between psychological mindedness and the relevant concepts presented in studies to determine the expected strength of the relationship. Where concepts appeared to have a moderate to strong relationship with psychological mindedness, for example alexithymia and other measures of psychological mindedness, the authors determined an expected strength of relationship of >0.50. For concepts with slight overlap with psychological mindedness, the authors determined an expected strength of relationship of >0.30, as suggested within COSMIN guidelines (Mokkink, Prinsen, et al. 2018).

## 3 Results

**FIGURE 2:**
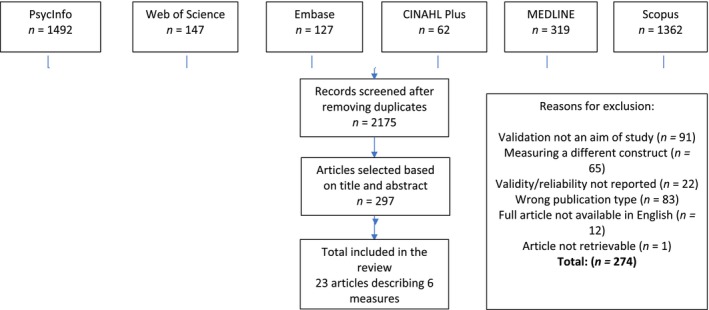
COSMIN flow chart (COSMIN 2017).

Database searches identified 2203 articles after duplicates were removed. Three hundred and twenty‐five articles were identified for full text review (see Figure 2). Of these, 23 studies relating to six measures of psychological mindedness were deemed to meet the eligibility criteria for the review. Ten studies evaluated measures that had been translated into a different language to the original format. See Table 3 for overview of characteristics for included studies.

There were some notable exclusions from the review. The California Personality Inventory–Psychological Mindedness subscale (CPI‐PY; Gough and Bradley 2002) includes items pertaining to alertness, aspiration and intellectual ability which do not appear directly related to the concept of psychological mindedness. The CPI‐PY appears to have been developed to reflect traits typically demonstrated by psychology students as opposed to being developed from a clear conceptualisation of psychological mindedness (Gough and Bradley 2002). The psychometric properties for the CPI‐PY scale are also published in the scale's manual and not published in a peer reviewed journal and therefore did not meet the inclusion criteria for the review. The Psychological Mindedness Rating Scale, based on the Psychological Mindedness Speech Sample (Berry et al. 2008), was excluded as the authors did not distinctly aim to assess the psychometric properties of the scale in studies using the scale.

Of the measures included in the review, five measures were used to assess psychological mindedness in adults. One measure (Hatcher et al. 1990) assessed self and other‐oriented psychological mindedness in adolescents. Measures used a variety of methods to assess psychological mindedness. Two measures, the Balanced Index of Psychological Mindedness (BIPM; Nyklicek and Denollet 2009) and the Psychological Mindedness Scale (PMS; Conte et al. 1990) were self‐report questionnaires. Most studies included in the review (*n =* 16) evaluated these self‐report measures. The remaining four measures involved clinician or researcher‐rated methods to assess psychological mindedness. Of these, the Psychological Mindedness Assessment Procedure (PMAP; McCallum and Piper 1990) used a video and interview technique in which individuals are presented with videos of simulated interactions (scenarios) between a therapist and client. The individual being assessed is asked to give their impression of the client's difficulties and their response is rated for the level of psychological mindedness demonstrated in their response. Two measures, Hatcher et al.'s (1990) measure of self and other‐oriented psychological mindedness in adolescents and Wolitzky and Reuben's measure of psychological mindedness used a thematic apperception test (TAT) technique. The TAT technique involves the presentation of stories and a clinician rating psychological mindedness based on the person's response to questions regarding the story. Dollinger et al.'s (1983) technique for measuring psychological mindedness involved measuring skill in Psychological Construing and Defence Understanding. Psychological Construing was measured through the description of personality and subsequent coding of level of psychological response. Defence Understanding was measured through presentation of vignettes describing a person employing defence mechanisms and subsequent rating of the person's response in describing the behaviour in the vignette.

Psychological mindedness has been described as both self‐oriented and other‐oriented. Of the six measures included in the review, the PMS (Conte et al. 1990), Hatcher et al.'s (1990) adolescent measure and Wolitzky and Reuben's (1974) TAT method reflected both self and other‐oriented psychological mindedness. One measure (BIPM; Nyklicek and Denollet 2009) reflected only self‐oriented psychological mindedness. The PMAP (McCallum and Piper 1990) appeared to only assess other‐oriented psychological mindedness. Dollinger et al.'s (1983) measure appeared to largely reflect other‐oriented psychological mindedness.

### 3.1 Reporting of Results

The overall outcome and quality of evidence for each measurement property of the measures is presented in Table 4. Results have been pooled where the methodology and statistical testing across studies are consistent. For example, where there are multiple studies indicating a high level of internal consistency, results are reported as *α* = >0.70. Where methods and statistical tests are not consistent across studies, results are presented separately. For construct validity, where studies have presented tests of multiple measures or outcomes, these have been treated as individual studies to determine the methodological quality of each measure and statistical analysis. For example, there are multiple cases in which a study has been rated as adequate quality for one test, and doubtful for a separate test. This is reflected in the overall quality of evidence. The measurement properties for content validity, cross‐cultural validity, measurement error and responsiveness are not presented as no studies evaluated these properties. Criterion validity is not presented because, as stated previously, the authors felt unable to determine a gold standard for the measurement of psychological mindedness. The quality and evidence of studies investigating the relationship between multiple measures of psychological mindedness were therefore evaluated using the hypothesis testing for construct validity measurement property, as per COSMIN guidelines (Mokkink, de Vet, et al. 2018).

### 3.2 Structural Validity

Only the BIPM (Nyklicek and Denollet 2009) met the COSMIN criteria for structural validity. Five studies evaluated this measurement property with consistent results presenting a two‐factor model of ‘insight’ and ‘interest’, reflecting the two subscales of the measure. Four studies presented either CFI = >0.95 OR RMSEA = <0.06 (Denizli et al. 2022; Giromini et al. 2015; Nyklicek and Denollet 2009; Takagishi 2020). One study evaluated the structure of the scale using principal component analysis which also supported the two‐factor structure (Amiruddin et al. 2021). The quality of this evidence was rated as high using the GRADE approach which increases confidence in the validity of the two‐factor structure.

The evidence for the structural validity for the PMS (Conte et al. 1990) was deemed indeterminate. The results of individual studies were inconsistent with two, four and five factor models for the PMS reported (Andelkovic 2020; Conte et al. 1990; Sahin and Yeniçeri 2015; Shill and Lumley 2002; Takagishi et al. 2014), therefore not meeting the COSMIN criteria for sufficiency of 75% of studies reporting the same factor structure (Mokkink, Prinsen, et al. 2018). Only one of these studies reported results which met the COSMIN criteria for sufficient measurement properties of RMSEA = <0.06 (Takagishi et al. 2014) in which a four‐factor structure of the PMS was reported. The quality of this study was graded as ‘adequate’, therefore providing the most robust evidence of the measure's factor structure. The evidence from the remaining four studies was deemed indeterminate due to confirmatory factor analysis not being conducted. The overall quality of evidence relating to the factor structure of the PMS was graded as ‘low’ due to multiple studies of doubtful quality and inconsistency in results.

### 3.3 Internal Consistency

Studies reported on the internal consistency of three measures: the BIPM, the PMS and Wolitzky and Reuben's TAT method.

Multiple studies of very good quality reported a sufficient level of internal consistency for the total scale and subscales of the BIPM (Amiruddin et al. 2021; Denizli et al. 2022; Giromini et al. 2015; Nyklicek and Denollet 2009; Takagishi 2020). These findings are consistent with the high quality of evidence relating to the two‐factor structure of the BIPM.

Where the PMS was evaluated as a unidimensional scale studies reported high internal consistency (Andelkovic 2020; Beitel et al. 2005; Conte et al. 1990; Conte et al. 1996; Hua et al. 2007; Sahin and Yeniçeri 2015; Shill and Lumley 2002; Takagishi et al. 2014). Studies evaluating internal consistency of subscales reported inconsistent results (Beitel et al. 2004; Hua et al. 2007; Sahin and Yeniçeri 2015). The overall quality of studies reporting evidence for the internal consistency of the PMS was rated as moderate quality using the GRADE approach. However, the indeterminate factor structure of the PMS limits the conclusions that can be drawn about the internal consistency of the scale as evaluation of internal consistency of a scale and subscales relies on sufficient evidence of the structure of the scale (Mokkink, de Vet, et al. 2018). The internal consistency of the TAT method (Wolitzky and Reuben 1974) was evaluated by one study of doubtful quality (Wolitzky and Reuben 1974). The evidence was deemed indeterminate due to the methodological limitation of using correlation analysis between individual items and total scale score as opposed to reporting Cronbach's alpha, as well as a lack of any studies reporting on the structural validity of the measure.

### 3.4 Reliability

Studies reported on the test–retest or inter‐rater reliability of all six measures included in the review.

#### 3.4.1 Test–Retest Reliability

Studies reported on the test–retest reliability of the BIPM, the PMAP and the PMS.

Four studies reported test–retest reliability of the BIPM (Denizli et al. 2022; Giromini et al. 2015; Nyklicek and Denollet 2009; Takagishi 2020). The evidence for the BIPM was deemed indeterminate due to unspecified details of statistical analysis in two studies (Denizli et al. 2022; Nyklicek and Denollet 2009) and insufficient statistical analysis (Pearson's correlation) in one study (Takagishi 2020). One study (Giromini et al. 2015) reported conducting adequate statistical analysis using intraclass correlation coefficient with insufficient reliability reported when measured against the COSMIN good measurement properties criteria (total scale: ICC = 0.61; insight subscale: ICC = 0.60; interest subscale: ICC = 0.50). However, the overall quality of the studies reporting on test–retest reliability was rated as ‘low’, limiting the conclusions that can be drawn about the test–retest reliability of the BIPM.

Two studies presented in one article reported on the test–retest reliability of the PMAP (McCallum and Piper 1990). The evidence for the test–retest reliability of the PMAP was deemed to be indeterminate according to the COSMIN good measurement properties criteria as the authors reported conducting Pearson's correlation rather than intraclass correlation coefficient in the studies (*r =* 0.59–0.76).

Two studies reported on the test–retest reliability of the PMS (Conte et al. 1996; Takagishi et al. 2014). Correlation coefficients were reported of 0.92 (Conte et al. 1996) and 0.71 (Takagishi et al. 2014). However, it is unclear whether the authors conducted intraclass correlation coefficient analyses or an alternative correlation coefficient such as a Pearson's correlation. Therefore, the studies did not meet the standard for sufficiency in reporting reliability according to the COSMIN guidelines for good measurement properties (Table 2; Mokkink, Prinsen, et al. 2018). As such, the evidence of test–retest reliability for the PMS was rated as indeterminate. The overall quality of the studies reporting the test–retest reliability of the PMS was deemed to be ‘moderate’ due to the evidence base of two studies of doubtful quality.

**TABLE 2:** COSMIN criteria for good measurement properties (Mokkink, Prinsen, et al. 2018).

Measurement property	Rating	Criteria
Structural validity	+	**CTT:** CFA: CFI or TLI or comparable measure >0.95 OR RMSEA <0.06 OR SRMR <0.08^2^ **IRT/Rasch**: No violation of unidimensionality^3^: CFI or TLI or comparable measure >0.95 OR RMSEA <0.06 OR SRMR <0.08 *AND* no violation of local independence: residual correlations among the items after controlling for the dominant factor <0.20 OR Q3's < 0.37 *AND* no violation of monotonicity: adequate looking graphs OR item scalability >0.30 *AND* adequate model fit: IRT: *χ* ^2^ > 0.01 Rasch: infit and outfit mean squares ≥0.5 and ≤1.5 OR *Z*‐standardised values > −2 and <2
	?	CTT: Not all information for ‘+’ reported IRT/Rasch: Model fit not reported
	−	Criteria for ‘+’ not met
Internal consistency	+	At least low evidence^4^ for sufficient structural validity^5^ AND Cronbach's alpha(s) ≥ 0.70 for each unidimensional scale or subscale^6^
	?	Criteria for ‘At least low evidence^4^ for sufficient structural validity^5^’ not met
	−	At least low evidence^4^ for sufficient structural validity^5^ AND Cronbach's alpha(s) < 0.70 for each unidimensional scale or subscale^6^
Reliability	+	ICC or weighted Kappa ≥ 0.70
	?	ICC or weighted Kappa not reported
	−	ICC or weighted Kappa < 0.70
Measurement error	+	SDC or LoA < MIC^5^
	?	MIC not defined
	−	SDC or LoA > MIC^5^
Hypotheses testing for construct validity	+	The result is in accordance with the hypothesis^7^
	?	No hypothesis defined (by the review team)
	−	The result is not in accordance with the hypothesis^7^
Cross‐cultural validity/measurement invariance	+	No important differences found between group factors (such as age, gender and language) in multiple group factor analysis OR no important DIF for group factors (McFadden's *R* ^2^ < 0.02)
	?	No multiple group factor analysis OR DIF analysis performed
	−	Important differences between group factors OR DIF was found
Criterion validity	+	Correlation with gold standard ≥ 0.70 OR AUC ≥ 0.70
	?	Not all information for ‘+’ reported
	−	Correlation with gold standard < 0.70 OR AUC < 0.70
Responsiveness	+	The result is in accordance with the hypothesis^7^ OR AUC ≥ 0.70
	?	No hypothesis defined (by review team)
	−	The result is not in accordance with the hypothesis OR AUC < 0.70

Abbreviations: AUC = area under the curve, CFA = confirmatory factor analysis, CFI = comparative fit index, CTT = classical test theory, DIF = differential item functioning, ICC = intraclass correlation coefficient, IRT = item response theory, LoA = limits of agreement, MIC = minimal important change, RMSEA = Root Mean Square Error of Approximation, SEM = Standard Error of Measurement, SDC = smallest detectable change, SRMR = Standardized Root Mean Residuals, TLI = Tucker‐Lewis index, ‘+’ = sufficient, ‘−’ = insufficient, ‘?’ = indeterminate.

**TABLE 3:** Study and measure characteristics.

Author, date and country	Measure	Subscales and/or domains assessed	Description	Sample size	Population	Psychometrics reported
Amiruddin et al. 2021, Malaysia	BIPM—Malaysian	Insight and interest	14‐item self‐report scale (7 = insight; 7 = interest). Self‐oriented (i.e., questions relating to the self rather than other)	141	Majority university students, *f* = 81, *m* = 62, age = 18 + mean age = not reported	Structural validity Internal consistency Construct validity
Andelkovic 2020, Serbia	PMS—Serbian	Willingness, openness, access to feelings, belief in benefits of discussing problems and interest	45‐item self‐report scale with self and other‐oriented items	166	University students, *f* = 145 m = 21, mean age = 21 (range = 19–30)	Structural validity Internal consistency Construct validity
Beitel and Cecero 2003, USA	PMS	See above	See above	187	Undergraduate students, *f* = 135 *m* = 52, mean age = 19.5 (SD = 3.50)	Construct validity
Beitel et al. 2004, USA	PMS	See above	See above	200	Undergraduate students, *f* = 155 *m* = 45, mean age = 26 (SD = 9.70)	Internal consistency Construct validity
Beitel et al. 2005, USA	PMS	See above	See above	103	Undergraduate students, *f* = 79 *m* = 24, mean age = 27 (SD = 8.80)	Internal consistency Construct validity
Conte et al. 1990, USA	PMS	See above	See above	44	Outpatients receiving therapy, *f* = 26 *m* = 18, mean age = 36.24 (SD = 13.55)	Measure development Internal consistency Construct validity
Conte et al. 1995, USA	PMS	See above	See above	46	Medical students, *f* = 25 *m* = 21, mean age = 24.8 (SD = 2.43)	Construct validity
Conte et al. 1996, USA	PMS	See above	See above	256	Psychiatric patients attending clinic, *f* = 145 *m* = 105, mean age = 36.37 (SD = 11.13)	Structural validity Internal consistency Reliability Construct validity
Denizli et al. 2022, Turkey	BIPM—Turkish	See above	See above	654	Undergraduate students, *f* = 434 *m* = 158, age range = 18–36	Structural validity Internal consistency Reliability Construct validity
Dollinger et al. 1983, USA		Psychological construing (PC) and defence understanding (DU)	Clinician‐rated measure based on responses to person descriptions (2‐point scale) and vignettes (3‐point scale)	70	College students, *f* = 31 m = 39, age = not reported	Measure development Reliability Construct validity
Giromini et al. 2015, Italy	BIPM—Italian	See above	See above	Study 1: 298 Study 2: 58 Study 3: 60	Study 1: Psychology students, *f* = 197 *m* = not reported, mean age = 21.3 (SD = 2.8) Study 2: psychology students *f* = 40 *m* = not reported, mean age = 21.7 (SD = 2.1) Study 3: Inpatient psychiatric = 30, *f* = 24, *m* = 6, mean age = 56.0 (SD = 9.6), non‐clinical sample = 30, *f* = 22 *m* = 8, mean age = 55.3 (SD = 9.8)	Structural validity Internal consistency Reliability Construct validity
Hatcher et al. 1990, USA	Adolescent measure	PM to self (PS) and PM to others (PO)	Clinician‐rated measure of responses to stories using a 4‐point Likert scale (PO) and 6‐point Likert scale (PS)	179 total	Elementary school children, Grade 5: *n* = 60, *f* = 29 m = 31, mean age 10.4. Grade 8: *n* = 60, *f* = 31 *m* = 29, mean age = 13.6. Grade 12: *n* = 59, *f* = 33 *m* = 26, mean age = 17.7	Measure development Reliability Construct validity
Hua et al. 2007, China	PMS—Chinese (Mandarin)	See above	See above	342	Mandarin speaking university students, *f* = 131 m = 211, mean age = 20.22 (SD = 1.39)	Structural validity Internal consistency Construct validity
McCallum and Piper 1990, Canada	PMAP	PM to other	Video and interview. Clinician‐rated. 9‐point scale indicating PM.	Study 1: 30 Study 2: 79	Study 1: non‐clinical adult sample (*n* = 30), *f* = 19 m = 11, mean age = 36.3 (range = 19–67) Study 2: psychiatric outpatients (*n =* 79), *f* = 53 m = 26, mean age = 35.7 (range = 18–65)	Measure development Reliability Construct validity
McCallum et al. 1992, Canada	PMAP	See above	See above	154 (109 after attrition)	Psychiatric outpatients, *f* = 72% *m* = not reported, mean age = 36	Construct validity
Nyklicek and Denollet 2009, Netherlands	BIPM	See above	See above	Study 1: 545 (533 after attrition) Study 2: 575 Study 3: 103	Study 1: Nonclinical adults. Sample 1: *n* = 333, *f* = 46% *m* = not reported, mean age = 49.1 (SD = 7.5). Sample 2: *n =* 200, *f* = 55% *m* = not reported, mean age = 33.4 (SD = 14.1) Study 2: Nonclinical sample, *f* = 55% *m* = not reported, mean age = 55.2 (SD = 14.6) Study 3: psychological recovery inpatients, *f* = 76% *m* = not reported. Mean age = 45.44 (SD = 9.98).	Measure development Structural validity Internal consistency Reliability Construct validity
Sahin and Yeniçeri 2015, Turkey	PMS—Turkish	See above	See above	418	University students, *f* = 299 *m* = 119, mean age = 20.38 (SD = 1.58)	Structural validity Internal consistency Construct validity
Segaar et al. 2023, Netherlands	PMAP—Dutch	See above	See above	194	Personality disorder diagnosis receiving intervention, *f* = 149 *m* = 45, mean age = 23.5 (SD = 4.5)	Reliability
Shill and Lumley 2002, USA	PMS	See above	See above	397	Undergraduate psychology students, *f* = 246 *m* = 144, mean age = 19.31 (range = 17–40)	Structural validity Internal consistency Reliability Construct validity
Smith et al. 2009, Netherlands	PMAP—Dutch	See above	See above	100	University students, *f* = 71 m = 29, mean age = 21.4 (range = 18–34)	Reliability Construct validity
Takagishi et al. 2014, Japan	PMS—Japanese	See above	See above	606	College students, *f* = 433 m = 173, mean age = 19.3 (SD = 3.5)	Structural validity Internal consistency Construct validity
Takagishi 2020, Jaoan	BIPM—Japanese	See above	See above	Study 1: 464 Study 2: 271	Study 1: university students, *f* = 228 *m* = 236, mean age = 19.4 (SD = 1.7) Study 2: university students, *f* = 186 *m* = 85, mean age = 19.9 (SD = 3.7)	Structural validity Internal consistency Reliability Construct validity
Wolitzky and Reuben 1974, USA	TAT story method	Predominantly PM to other (nine items), with one item appearing to reflect PM to self (self‐awareness)	Clinician‐rated measure. 40‐point scale based on interview responses.	14	Undergraduate students, *m* = 14 age not reported	Internal consistency Reliability Construct validity

*Note:* BIPM: Balanced Index of Psychological Mindedness; DU: Defence Understanding; *f*: females; *m*: males; PC: Psychological Construing; PM: Psychological Mindedness; PO: Psychological Mindedness to Others; PS: Psychological Mindedness to the Self; PMAP: Psychological Mindedness Assessment Procedure; PMS: Psychological Mindedness Scale; SD: standard deviation; TAT: Thematic Apperception Test.

**TABLE 4:** Summary of pooled results.

	Description of results	Overall rating	Quality of evidence
**Structural validity**			
BIPM	Two factor model confirmed (CFI = 0.86–0.955)	Sufficient (+)	High Five studies of very good quality
PMS	Five factor model not confirmed Four factor model found (RMSEA = 0.50)	Indeterminate (?)	Low Multiple studies of doubtful quality, inconsistent findings
**Internal consistency**			
BIPM	Total: *α* = >0.70. insight: *α*= >0.70 interest: *α*= >0.70 and high quality of evidence of structural validity	Sufficient (+)	High Multiple studies of very good quality
PMS	Unidimensional scale: *α* = >0.70; Factorial *α* = 0.51–0.80 with inconsistent findings of structural validity	Indeterminate (?)	Moderate Multiple studies of doubtful quality
TAT method	Item correlation with total score range: *r =* 0.33–0.83 with no evidence of structural validity	Indeterminate (?)	Very low One study of doubtful quality
**Reliability**			
Adolescent PO/PS measure	PO: *k* = 0.48–0.89 PS: *k* = 0.69–0.79	Insufficient (−)	Very low One study of doubtful quality
BIPM	Total: (assumed) *r* = <0.70; ICC = 0.61 Insight: (assumed) *r =* <0.70; ICC = 0.60 Interest: (assumed) *r* = <0.60; ICC = 0.50	Indeterminate (?)	Low Multiple studies of doubtful quality
Psychological construing and defence understanding	PC: *r* = 0.82 DU: *r* = 0.70	Indeterminate (?)	Very low One study of doubtful quality
PMAP	Total: ICC = 0.69–0.96; *r =* 0.59–0.76 Individual scenarios: ICC = 0.46–0.82	Inconsistent (+/−)	Low Multiple studies of doubtful quality
PMS	Total*: r =* 0.72–0.92 Factors range: *r =* 0.68–0.74	Indeterminate (?)	Moderate Two studies of doubtful quality
TAT method	*r =* 0.96	Indeterminate (?)	Low One study of doubtful quality
**Hypothesis testing for construct validity**			
Adolescent PO/PS measure	2 out of 5 hypotheses confirmed	Insufficient (−)	Very low Multiple cases of doubtful quality
BIPM	18 out of 26 hypotheses confirmed	Insufficient (−)	High Multiple studies of adequate quality
Psychological construing and defence understanding	0 out of 7 hypotheses confirmed	Insufficient (−)	Very low Multiple cases of inadequate quality
PMAP	4 out of 12 hypotheses confirmed	Insufficient (−)	Low Multiple studies of doubtful quality
PMS	23 out of 47 hypotheses confirmed	Insufficient (−)	High Multiple studies of at least adequate quality
TAT method	2 out of 2 hypotheses confirmed	Sufficient (+)	Very low One study of inadequate quality

#### 3.4.2 Inter‐Rater Reliability

Studies reported on the inter‐rater reliability of the Psychological Construing and Defence Understanding method (Dollinger et al. 1983), the PMAP (McCallum and Piper 1990; Segaar et al. 2023; Smith et al. 2009) and the TAT method (Wolitzky and Reuben 1974). Hatcher et al. (1990) reported on the reliability of the adolescent PO and PS measures, which was assumed to be inter‐rater reliability, although this is not explicitly stated.

One study reported on the inter‐rater reliability of the Psychological Construing (*r =* 0.82) and Defence Understanding (*r =* 0.70) method (Dollinger et al. 1983). The evidence for the inter‐rater reliability of the method is deemed indeterminate according to COSMIN good measurement properties due to the authors conducting a Pearson's correlation. The quality of the evidence was rated as very low due to one study of doubtful quality, limiting the conclusions that can be drawn about the inter‐rater reliability of the method.

Four studies in three papers reported on the inter‐rater reliability of the PMAP (McCallum and Piper 1990; Segaar et al. 2023; Smith et al. 2009). Three papers reported inter‐rater reliability of the total PMAP and two papers reported on individual scenarios presented in the PMAP. The evidence for the inter‐rater reliability of the total PMAP was deemed to be insufficient due to two of the three studies reporting ICC < 0.70. Evidence of the inter‐rater reliability of individual scenarios of the PMAP was also deemed insufficient based on the level of correlation reported (ICC = 0.36–0.82). The overall quality of studies reporting on inter‐rater reliability of the PMAP was deemed to be low with multiple studies of doubtful quality.

One study reported on the inter‐rater reliability of the TAT method (Wolitzky and Reuben 1974). The evidence was deemed indeterminate due to the statistical analysis using Pearson's correlation (*r =* 0.96). The quality of the evidence for the inter‐rater reliability of the TAT method was graded as ‘low’ due to only one study of doubtful quality, limiting conclusions that can be drawn regarding the inter‐rater reliability of the TAT method.

One study reported on the reliability of the adolescent PO and PS measures (Hatcher et al. 1990). The evidence presented was deemed insufficient due to inconsistent reliability reported across various cases in the study for the PO (*k* = 0.48–89) and PS (*k* = 0.69–0.79) measures. The quality of the overall evidence was graded as low due to one study of doubtful quality and inconsistency in level of reliability reported.

Overall, the sufficiency of evidence relating to the test–retest and inter‐rater reliability of all measures included in the review was insufficient or indeterminate with the quality of evidence for all but one measure (PMAP) graded as ‘low’.

### 3.5 Hypothesis Testing for Construct Validity

Studies reported evidence relating to construct validity for all six measures included in the review. The overall evidence relating to measures was only deemed sufficient for the TAT method (Wolitzky and Reuben 1974) with two out of two hypotheses confirmed. However, the quality of this evidence was deemed very low with only one study of inadequate quality conducted due to lack of evidence for the psychometric properties of comparator instruments. The overall evidence for the adolescent measure of PO and PS (40% of hypotheses confirmed), the BIPM (69%), Psychological Construing and Defence Understanding (0%), the PMAP (33%) and the PMS (49%) was deemed insufficient when evaluated against the COSMIN good measurement properties (Mokkink, Prinsen, et al. 2018) with less than 75% of hypotheses confirmed for each measure. Eighteen out of 26 (69%) hypotheses were confirmed for studies evaluating the BIPM. The overall quality of this evidence was high with multiple studies of at least adequate quality. Of note, the BIPM was found to be negatively associated with alexithymia in four studies (*r =* −0.38–0.64) indicating that some items of the BIPM may reflect an absence of alexithymia, though one of these studies reported a strength of relationship lower than the expected strength of relationship of *r =* −0.50 determined by the authors. The BIPM was found to be positively associated with mindfulness in two studies (*r =* 0.09–0.50). The BIPM was found to be positively associated with emotional awareness through measurement of the overall and subscales of the Trait Meta Mood Scale (*r* = 0.28–0.70). In one study, the BIPM was positively associated with public and private self‐consciousness (*r =* 0.39; *r =* 0.57). The BIPM was also positively associated with the PMS in two studies (*r =* 0.40–0.63) though one of these studies reported a strength of relationship lower than the relationship expected of *r =* 0.50 determined by the authors. For the PMS, 23 out of 47 (49%) hypotheses were confirmed. The overall quality of the evidence evaluating the construct validity of the PMS was deemed to be high with multiple studies of at least adequate quality. Of note, the PMS was negatively associated with alexithymia (*r* = −0.31–0.67) in two studies. One of these studies reported a strength of relationship below the expected strength of relationship (*r =* −0.50) determined by the authors. The PMS was positively associated with mindfulness in one study (*r =* 0.40). One study measured this relationship but did not report the results in English, therefore the authors were unable to determine the results of the study (Sahin and Yeniçeri 2015). The PMS was positively associated with private self‐consciousness (*r =* 0.27) and a slight negative association with public self‐consciousness was reported (*r =* −0.04). The PMS was negatively associated with depression in one study (*r* = −0.27). Of note, two studies reported on the relationship between the PMAP and three measures which have been associated with the concept of psychological mindedness but did not meet the inclusion criteria for this study. The PMAP was found to be positively associated (*r =* 0.42) with the California Personality Inventory (PY subscale; Gough and Bradley 2002), the Insight Test (Tolor and Reznikoff 1960; *r =* 0.50) and subjective clinical judgement of psychological mindedness (*r =* 0.30). This indicates that the PMAP may demonstrate a level of convergent validity with other measures associated with psychological mindedness though the psychometric rigour of these measures is unclear and the evidence for this relationship is therefore rated as doubtful quality.

#### 3.5.1 Discriminant Validity

Three studies included in the review investigated differences between sub‐groups. Two studies investigated differences between sub‐groups using the BIPM. Giromini et al. (2015) investigated differences in BIPM scores between clinical and non‐clinical samples. A medium effect size was reported for differences between clinical and non‐clinical groups for BIPM total (*d* = 0.71) and BIPM interest (*d =* 0.58). A large effect of greater than one standard deviation was reported for difference in BIPM insight (*d =* 1.76) between clinical and non‐clinical groups. Nyklicek and Denollet (2009) investigated differences in BIPM scores between patient and community samples. A large effect size greater than one standard deviation was reported for differences in total BIPM scores between patient and community samples (*d =* 1.10). The results from both studies met the author's hypothesis of a minimum medium effect (*d =* >0.50) for group comparisons.

One study included in the review reported investigating differences between remainers and drop‐outs in psychological therapy using the PMAP (McCallum et al. 1992). McCallum et al. (1992) reported a medium effect size for differences in PMAP scores between remainers and drop‐outs (*d =* 0.66) which met the authors' criteria of (*d =* >0.50) for group differences.

### 3.6 Synthesis

An overview of the findings for each measurement property of each measure included in the review is presented in Table 4. Overall, no measure included in the review demonstrated sufficiency across all measurement properties. Based on studies included in this review, the BIPM (Nyklicek and Denollet 2009) appeared to have the most credible evidence base. The BIPM was found to have sufficient evidence of structural validity and internal consistency with high quality evidence presented. The BIPM also had the highest proportion of hypotheses confirmed for construct validity with high quality evidence presented, albeit insufficient overall when evaluated using the COSMIN guidelines for good measurement properties with less than 75% of hypotheses confirmed. The measurement properties for the remaining five measures were less sufficient and with evidence presented by studies generally of lower quality. The reliability of the PMS (Conte et al. 1990) was found to be indeterminate with inconsistency in the quality of evidence presented. The validity of the PMS was found to be indeterminate or insufficient with an inconsistent level of quality of the evidence presented. The quality of evidence for the remaining four measures was of ‘low’ or ‘very low’ quality, making it difficult to draw conclusions regarding the psychometric properties of these measures.

## 4 Discussion

The review suggests that current measures of psychological mindedness are generally inadequate when they are assessed across a range of measurement properties relating to reliability and validity. Whilst no measure performed well across all measurement properties, the BIPM demonstrated sufficient evidence for structural validity and internal consistency. The BIPM also performed best in hypothesis testing for construct validity, albeit still insufficient according to COSMIN guidelines with 69% of hypotheses meeting the minimum expected level of either the relationship between constructs or difference between subgroups set by the authors of this review. The BIPM can feasibly be administered in various clinical and research settings due to the relatively brief format of a 14‐item self‐report questionnaire. Studies have presented evidence for the measurement properties of the BIPM from various clinical and non‐clinical populations and in various languages with the psychometric properties appearing to remain relatively stable across these settings. However, it should be noted that no studies were identified that met the COSMIN criteria of cross‐cultural validation studies.

Clinicians and researchers using the BIPM should also consider which aspect of psychological mindedness they are most interested in. Various definitions of psychological mindedness have identified the concept as incorporating both psychological mindedness in relation to the self and in relation to others. Clinicians and researchers using the BIPM should note that the BIPM is largely ‘self‐oriented’ and was the only measure of psychological mindedness included in the review that does not incorporate an ‘other’ oriented element to the measure. Researchers and clinicians should therefore consider the appropriateness of the BIPM if they aim to capture the broader concept of psychological mindedness as the BIPM should not be used to measure other‐oriented aspects of psychological mindedness.

The PMS incorporates both ‘other’ and ‘self‐oriented’ aspects of psychological mindedness and may be more suitable for researchers and clinicians looking to measure the broader concept of psychological mindedness including psychological mindedness towards others. However, most studies evaluating the PMS included in this review evaluated the measure in student populations. This review did not find sufficient evidence of the validity and reliability of the PMS in clinical and non‐clinical general adult populations. The evidence for the validity and reliability of the PMS presented by studies included in this review demonstrated insufficient psychometric rigour of the PMS with indeterminate evidence of structural validity, internal consistency, and reliability, and insufficient evidence of construct validity when evaluated using the COSMIN approach.

Both the BIPM and PMS are self–reported measures of psychological mindedness. Some research has highlighted differences between self‐reported and clinician or researcher‐rated (observer) levels of psychological mindedness with weak and moderate correlations reported (Hartley et al. 2016; McCallum and Piper 1990). It has been suggested that independent ratings may be more accurate than self‐reported level of psychologist mindedness which are subject to reporting bias (Hartley et al. 2016). Self‐report measures of psychological mindedness assess the individual's interest in and personal impression of psychological mindedness which may be over and underestimated in individuals and within some clinical presentations. Observer‐reported measures of psychological mindedness may be helpful in developing an accurate impression of an individual's level of psychological mindedness alongside or instead of self‐report measures. Whilst the PMAP appears to have the largest evidence base for observer‐reported measures of psychological mindedness, researchers and clinicians who prefer to administer an observer‐reported measure are advised that evidence for observer‐reported measures in this review was not rated as sufficient.

The review found evidence of convergent validity between self‐report measures of psychological mindedness (Nyklicek and Denollet 2009; Takagishi 2020). However, the instruments included in this review varied in their orientation of psychological mindedness to the self and others. The measures also varied in incorporating different facets of psychological mindedness, including but not limited to interest and insight (Nyklicek and Denollet 2009) and willingness to understand others and access to feelings (Conte et al. 1996). These differences highlight the variance in how the multi‐faceted concept of psychological mindedness has been understood and operationalised. The differences in how the concept has been understood represents a barrier for operationalisation of psychological mindedness in clinical and research settings and limits interpretation of measures to the facets of psychological mindedness that a measure reflects (i.e., self‐oriented, other‐oriented, willingness and insight). This review highlights the need for further standardisation of the conceptual structure of psychological mindedness and the working definition derived for the purpose of our review may provide a good starting point.

Psychological mindedness has been positively associated with mental wellbeing (Trudeau and Reich 1995) and has been discussed as a trait that can support engagement and benefit from psychological therapy (McCallum and Piper 1997; Trudeau and Reich 1995). Two studies included in the review investigated differences in psychological mindedness between clinical and non‐clinical populations as measured by the BIPM, finding significant differences between the groups (Giromini et al. 2015; Nyklicek and Denollet 2009). A medium effect size for differences between groups was reported within these studies which exceeded the minimum expected difference between groups set by the authors of this review (*d =* >0.50). One study in this review (McCallum et al. 1992) found significant differences in psychological mindedness as measured by the PMAP between individuals who remained in therapy compared to individuals that dropped out. The results from this study also exceeded the minimum expectation for differences between groups set by the authors of this review. There is a need for further exploration of a causal relationship between psychological mindedness and psychological wellbeing. However, the findings from Nyklicek and Denollet (2009) may suggest that psychological mindedness can promote psychological health through a person's interest in understanding their thoughts and feelings and in turn developing understanding regarding their internal experiences. In this sense, a higher level of psychological mindedness may serve as a buffer against distress and increase likelihood of remaining in psychological therapy (McCallum et al. 1992).

Psychological mindedness has been considered in relation to various similar constructs. Six studies investigated the relationship between psychological mindedness and alexithymia using either the BIPM (*n =* 4) or the PMS (*n* = 2). Alexithymia was measured using the Toronto Alexithymia Scale‐20 (TAS‐20; Bagby et al. 1994) in all six studies. All six hypotheses were confirmed with all studies rated as having either adequate or high methodological quality. Both the BIPM (*r =* −0.38–0.64) and the PMS (*r =* −0.31–67) demonstrated a similar strength of negative relationship across multiple studies. As such, this review provides strong evidence for the discriminant relationship between psychological mindedness and alexithymia with both the BIPM and PMS demonstrating a negative relationship with the TAS‐20.

Four studies investigated the relationship between psychological mindedness and mindfulness using either the BIPM (*n* = 2) or the PMS (*n* = 2). Two out of three hypotheses were confirmed with the overall evidence base reporting on these results as of ‘moderate’ quality when rated using the GRADE approach. One study (Sahin and Yeniçeri 2015) did not report the results of their analysis in English and therefore the authors were unable to interpret the results of this study. The BIPM was found to have a varying degree in the strength of relationship with mindfulness (*r =* 0.09–0.50). The BIPM appeared strongly related to the Five Facets Mindfulness Questionnaire (Baer et al. 2006) but a minimal relationship was found with the Mindfulness, Awareness and Attention Scale (MAAS; Brown and Ryan 2003). A moderate strength of positive relationship was found between the PMS and the MAAS (*r =* 0.41). Overall, this review indicates a positive relationship between psychological mindedness and mindfulness, though it should be noted that one study of adequate quality (Amiruddin et al. 2021) did not report a relationship between psychological mindedness and mindfulness indicating some inconsistency in results exploring this relationship.

Two studies in the review investigated the relationship between psychological mindedness and aspects of self‐consciousness including ‘private’ and ‘public’ self‐consciousness, and reflection and rumination which have been associated with private self‐consciousness (Harrington and Loffredo 2011). The concepts of psychological mindedness and private self‐consciousness have previously been used synonymously (Farber 1989), and it has been argued that measures relating to private self‐consciousness, such as self‐reflection and insight, can be used to measure psychological mindedness (Grant et al. 2002). However, Grant (2001) proposed that private self‐consciousness which involves examination of one's mental and emotional processes may be one of various concepts that combine to form psychological mindedness. Within this review, a study using the BIPM (Nyklicek and Denollet 2009) indicated a positive relationship with private and public self‐consciousness. The BIPM was found to have a strong relationship with private self‐consciousness and a moderate strength relationship with public self‐consciousness. A study using the PMS (Beitel et al. 2005) found a positive relationship with private self‐consciousness and a slightly negative relationship with public self‐consciousness, though the strength of these relationships were not to the level expected by the authors of this review. However, previous findings from Trudeau and Reich (1995), a study which did not meet eligibility for the current review, reported a moderate positive relationship between the PMS and private self‐consciousness. The results from the small number of studies included in this review that explored this relationship indicated that whilst psychological mindedness and private self‐consciousness may be positively associated, the relationship is inconsistent and the two concepts should not be used synonymously. Clinicians and researchers should exercise caution in drawing conclusions regarding a person's level of psychological mindedness when using measures related to private self‐consciousness.

Taken as a whole, the evidence in this review of a negative relationship with alexithymia and the positive relationship with mindfulness supports the notion that psychological mindedness may support psychological wellbeing.

### 4.1 Recommendations for Future Research

The evidence base for the psychometric properties of measures of psychological mindedness would benefit from further research into the content validity, cross‐cultural validity, measurement error and responsiveness of all measures included in the review. With evidence that a person's level of psychological mindedness can change following psychological therapy (Nyklicek et al. 2010), future studies investigating the responsiveness of measures would support appropriate use of measures of psychological mindedness as an outcome measure in psychological therapy. The BIPM was evaluated as having the strongest psychometric properties yet does not incorporate ‘other‐oriented’ psychological mindedness, a feature often included in definitions and conceptual overviews of psychological mindedness. Future research should therefore focus on evaluation of the measurement properties of measures incorporating an ‘other‐oriented’ dimension of psychological mindedness such as the PMS or Psychological Mindedness Rating Scale (PMRS; Berry et al. 2008). The PMRS, which was not evaluated in this review, has demonstrated reliability in the measurement of clinicians' level of psychological mindedness in a small number of studies in mental health settings (Berry et al. 2008; Bourne et al. 2014; Hartley et al. 2016). The measure incorporates a strong emphasis on ‘other‐oriented’ psychological mindedness through clinicians' consideration of service users' experiences. The PMRS is an observer‐reported measure of psychological mindedness and if used by clinicians and researchers may be helpful in developing an accurate impression of an individual's level psychological mindedness as opposed to relying on self‐report measures which may be subject to bias. The PMRS may be suitable for use instead of or alongside the BIPM in research aiming to evaluate psychological mindedness towards others. Future research should therefore aim to further investigate the reliability and validity of this measure in other settings. Greater conceptual understanding of psychological mindedness would be supported by further research investigating the distinct convergent and discriminant relationships of self and other oriented psychological mindedness with related concepts such as alexithymia, mentalization, mindfulness, self‐consciousness and with outcome measures such as depression, anxiety, and psychological wellbeing. For example, researchers may consider exploring how measures of self‐oriented (e.g., BIPM) and other‐oriented psychological mindedness (e.g., PMRS) are associated with other constructs in one sample. Conceptual understanding of psychological mindedness could be developed through future studies adopting consensus methods. For example, a multiple‐round Delphi study may help to generate consensus in an expert panel with regard to the definition and structure of a concept such as psychological mindedness (Armstrong et al. 2012; Muhl et al. 2023).

### 4.2 Limitations

The COSMIN guidance for systematic reviews was primarily developed to review the measurement properties of PROMs. The COSMIN methodology was used in this review as the majority of papers returned in the initial searches related to the evaluation of self‐report measures of psychological mindedness. However, it should be noted that four measures included in this review did not use a self‐report method to measure psychological mindedness. Some aspects relating to the validity and reliability of clinician‐reported outcomes, such as factors impacting inter‐rater reliability of measures including consistency in the approach of researchers in a study, are not formally evaluated within the COSMIN criteria. In this instance the authors of the review considered these factors under the ‘any other flaws in the design or statistical methods’ item of various measurement properties evaluated. However, there was limited guidance in evaluating this aspect of studies due to the absence of these factors in the formal COSMIN approach.

The eligibility criteria based on COSMIN guidance also specified including only studies which explicitly aim to evaluate the psychometric properties of a measure of psychological mindedness, with studies using a measure of psychological mindedness to validate a measure of another concept excluded from the review. However, it should be noted that this led to the exclusion of various studies that inadvertently reported evidence relevant to psychometric properties of the measures included. For example, the authors are aware of studies using measures included in this review to explore the relationship between psychological mindedness and other concepts such as private self‐consciousness (Trudeau and Reich 1995) and creative cognition (LeBoutillier and Barry 2018). These studies were not included in the review as the studies did not explicitly aim to assess the psychometric properties of the measures used in the studies. Similarly, measures included in this review have been used in studies of psychological mindedness in therapy settings to investigate change in psychological mindedness following therapy (Nyklicek et al. 2010) and the relationship between psychological mindedness and depression in therapy (Kronström et al. 2009). However, these studies, whilst relevant to the review topic, were excluded as they did not explicitly aim to investigate the psychometric properties of the measures. Therefore, this review may not be wholly representative of wider inadvertent evidence relating to psychometric properties of the measures included in the review.

There are also some limitations which apply to interpretation of the papers included in the review. The COSMIN methodology specifies certain statistical analyses in interpreting measurement properties. For example, the COSMIN methodology specifies only intraclass correlation coefficient or Cohen's kappa as a sufficient indicator of test–retest reliability. Studies reporting other statistical analyses such as Pearson's correlation, or studies lacking sufficient details regarding the model of intraclass correlation coefficient, are rated as insufficient according to the COSMIN good measurement properties criteria. This is despite some authors suggesting that single tests of reliability are not sufficient indicators of reliability (Bruton et al. 2000). For example, the authors draw attention to various studies reporting on the reliability of the BIPM which indicated moderate reliability of the measure but did not conduct or provide sufficient detail of a model of intraclass correlation coefficient (Denizli et al. 2022; Giromini et al. 2015; Nyklicek and Denollet 2009; Takagishi 2020). Evidence reported in these individual studies is presented in the Supporting Information.

## 5 Conclusion

The review indicates a lack of measures of psychological mindedness that demonstrate sufficient measurement properties across all domains of reliability, validity and responsiveness evaluated in this review. The BIPM was identified as having the strongest psychometric rigour, demonstrating sufficient high‐quality evidence of structural validity and internal consistency. However, sufficient evidence was lacking for the scale in other areas including test–retest reliability and construct validity. Further research is needed to evaluate other measurement properties of measures of psychological mindedness including the BIPM as no studies included in the review evaluated the content validity, cross‐cultural validity, measurement error or responsiveness of measures of psychological mindedness. Further research is needed to develop reliable and valid observer‐rated methods of measuring psychological mindedness to supplement self‐report measures.

## Conflicts of Interest

The authors declare no conflicts of interest.

## Supporting information


**Appendix SA** *Systematic review search terms.*
Appendix SB. *Results of individual studies from systematic review*.

## Data Availability

There is no primary data associated with this research.
