# Supplementary material for: Measures of Psychological Mindedness: A Systematic Review of Psychometric Properties
Source: Clin Psychol Psychother. 2025 Mar 23;32(2):e70064. doi: 10.1002/cpp.70064 (PMC11930890; doi:10.1002/cpp.70064)
Supplement: Supplementary file 1 — Appendix SA Systematic review search terms. Appendix SB. Results of individual studies from systematic review. [file CPP-32-e70064-s001.docx]

**Supplementary Materials – to be uploaded as separate files**

**Appendix A**

***Systematic review search terms***

(("psychological mindedness" or "psychological-mindedness" or "california personality inventory" or "insight test")

AND

(measure* or instrument* or interview* or assessment* or questionnaire*)).af.

**Appendix B**

***Results of individual studies from systematic review***

| **Structural validity** | | | | | |
| --- | --- | --- | --- | --- | --- |
| **Measure (author, date)** | **Language** | **N** | **Methodological quality** | **Result** | **Good measurement properties rating** |
| BIPM (Amiruddin et al. 2021) | Malaysian | 141 | Adequate | Two factor model (insight, interest) explaining 56.56% variance (Principal Component Analysis) | (-) |
| BIPM (Denizli et al. 2022) | Turkish | 654 | Very good | Two factor model (insight, interest) confirmed. CFI= .94; RMSEA= .060; SRMR= .059 | (+) |
| BIPM (Giromini et al. 2015) | Italian | 298 | Very good | Two factor model confirmed. CFI = .86; RMSEA= .085; SRMR= .07 |  |
| BIPM (Nyklicek & Denollet, 2009) | English | 1108 | Very good | Two factor model (insight, interest). Three factor analyses conducted. CFI = .895-.939; RMSEA=.043-.050. |  |
| BIPM (Takagishi, 2020) | Japanese | 271 | Very good | Two factor model confirmed (insight, interest). CFI=.955; RMSEA=.057 | (+) |
| PMS (Andelkovic, 2020) | Serbian | 166 | Inadequate | **PCA identified four factors:** 1) belief in the benefits of discussing one's problems 2) access to feelings 3) interest in the meaning and motivation of one's and others' behaviour 4) openness to new ideas. **32% variance explained** | (-) |
| PMS (Conte et al. 1990) | English | 44 | Inadequate | Five factor model proposed via correlation with therapeutic outcomes | (-) |
| PMS (Hua et al. 2007) | Mandarin | 342 | Inadequate | Not reported | (?) |
| PMS (Sahin et al. 2015) | Turkish | 418 | Doubtful | **PCA. Five factors:** 1) sharing 2) emotional awareness 3) willingness to understand the reasons under others' behaviour 4) closing oneself to change 5) openness to new information. **33.48% variance explained.** | (-) |
| PMS (Shill & Lumley, 2002) | English | 397 | Adequate | Did not support five/four factor model identified previously. **Two factors then five factors identified but different to those identified previously.** | (-) |
| PMS (Takagishi et al. 2014) | Japanese | 606 | Adequate | **EFA: Four factors:** 1) beliefs in the benefits of discussing one's problems 2) access to feelings 3) openness to new ideas and capacity for change 4) interest in the meaning and motivations of one's own and others' behaviour. **CFA: The final model is presented in Figure1, χ2(309) = 577.585; GFI = .915; AGFI = .892; RMSEA = .050; and AIC = 382.982. 42.4% variance explained** | (+) |
| **Internal Consistency** |  |  |  |  |  |
| BIPM (Amiruddin et al. 2021) | Malaysian | 141 | Very good | total: α=.87. insight: α=.87. interest: α=.82 total: omega= .88 | (+) |
| BIPM (Denizli et al. 2022) | Turkish | 654 | Very good | Total: .85 Insight: .76. Interest: .73 | (+) |
| BIPM (Giromini et al. 2015) | Italian | 298 | Very good | total: .76. insight: .79. interest: .78 | (+) |
| BIPM (Nyklicek & Denollet, 2009) (a) | English | 533 | Very good | Total: .85 Insight: .76. Interest: .85 | (+) |
| BIPM (Nyklicek & Denollet, 2009) (b) | English | 575 | Very good | Total: .87 Insight: .85. Interest: .83 | (+) |
| BIPM (Takagishi, 2020) | Japanese | 464 | Very good | Total: .819. Insight: .787 Interest: .778 | (+) |
| PMS (Andelkovic, 2020) | Serbian | 166 | Doubtful | α=.83 | (?) |
| PMS (Beitel et al. 2004) | English | 200 | Very good | Subscales (based on Shill & Lumleys FA: Beliefs α=.79  Feelings α=.67  Understand α=.67  Meaning α=.56  Openness α=.52 | (?) |
| PMS (Beitel et al. 2005) | English | 103 | Doubtful | α=.86 | (?) |
| PMS (Conte et al. 1990) | English | 44 | Doubtful | α=.86 | (?) |
| PMS (Conte et al. 1996) | English | 256 | Doubtful | α=.87 | (?) |
| PMS (Hua et al. 2007) | Mandarin | 342 | Very good | Main Scale: α=.73. | (?) |
| PMS (Sahin et al. 2015) | Turkish | 418 | Very good | Main Scale: α=.79.  Subscales range: α=.51 - .80 | (?) |
| PMS (Shill & Lumley, 2002) | English | 397 | Doubtful | α=.80 | (?) |
| PMS (Takagishi et al. 2014) | Japanese | 606 | Doubtful | α=.81 | (?) |
| TAT method (Wolitzky & Reuben, 1974) | English | 14 | Doubtful | Correlation of items with total score (range): .33-.83 | (?) |
| **Reliability** |  |  |  |  |  |
| Adolescent measure (Hatcher et al. 1990) | English | 179 | Doubtful | Inter-rater reliability: PO: Range .48 - .89 PS: Range: .69 - .79 | (?) |
| BIPM (Denizli et al. 2022) | Turkish | 654 | Adequate | Test-retest coefficient: Total: .61 Interest: .55 Insight: .56 | (?) |
| BIPM (Giromini et al. 2015) | Italian | 58 | Adequate | Test-retest: Total: ICC=.61 Interest: ICC=.50 Insight: ICC=.60 | (-) |
| BIPM (Nyklicek & Denollet, 2009) | English | 184 | Doubtful | Test-retest: Total: .75 Interest: .63 Insight: .71 | (?) |
| BIPM (Takagishi, 2020) | Japanese | 271 | Doubtful | Test-retest: Total: *r=*.63 Interest: *r=*.59 Insight: *r=*.59 | (?) |
| Psychological construing and defence understanding (Dollinger et al. 1983) | English | 70 | Doubtful | Inter-rater reliability: PC: *r* =.82; DU: *r* =.70 | (?) |
| PMAP (McCallum & Piper, 1990) (a) | English | 15 | Doubtful | Inter-rater reliability: ICC=.60  Test-retest: *r=*.76 | (-)  (?) |
| PMAP (McCallum & Piper, 1990) (b) | English | 15 | Doubtful | Inter-rater reliability: ICC=.96  Test-retest: *r(27)*=.59 | (+)  (?) |
| PMAP (Segaar et al. 2023) | Dutch | 194 | Very good | ICC= .59 | (-) |
| PMAP (Smith et al. 2009) | Dutch | 100 | Doubtful | ICC =.81; .82; .82; .50 | (+) |
| PMS (Conte et al. 1996) | English | 22 | Doubtful |  |  |
| PMS (Takagishi et al. 2014) | Japanese | 606 | Doubtful | Total*: r=*.72.  Factors range: *r=*.68-.74 | (?) |
| TAT method (Wolitzky & Reuben, 1974) | English | 14 | Doubtful | Inter-rater reliability: *r*=.96 | (?) |
| **Hypothesis testing for construct validity** |  |  |  |  |  |
| Adolescent measure (Hatcher et al. 1990) | English | 179 | Doubtful *n* = 5 | Convergent: 3 (-)  Discriminant: 2 (+) | 3 (-)  2 (+) |
| BIPM (Amiruddin et al. 2021) | Malaysian | 141 | Adequate *n* =2 | Convergent: 1 (+)  Discriminant: 1 (+) | 2 (+) |
| BIPM (Denizli et al. 2022) | Turkish | 654 | Adequate *n* = 1 | Discriminant: 1 (+) | 1 (+) |
| BIPM (Giromini et al. 2015) | Italian | 298 | Adequate *n* = 4  Doubtful *n* = 3 | Convergent: 3 (+)  Discriminant: 2 (+) 2 (-) | 5 (+)  2 (-) |
| BIPM (Nyklicek & Denollet, 2009) | English | 533 | Adequate *n* = 5  Doubtful *n* = 6 | Convergent: 6 (+) 1 (-)  Discriminant: 2 (+) 2 (-) | 7 (+)  3 (-) |
| BIPM (Takagishi, 2020) | Japanese | 271 | Adequate *n* = 3  Doubtful *n* = 1 | Convergent: 2 (-)  Discriminant: 2 (+) | 2 (+)  2 (-) |
| PMAP (McCallum & Piper, 1990) (a) | English | 30 | Doubtful *n* = 4 | Convergent: 1 (+) 1 (-)  Discriminant: 2 (?) | 1 (+)  2 (?)  1 (-) |
| PMAP (McCallum & Piper, 1990) (b) | English | 79 | Doubtful *n* = 6  Inadequate *n* = 1 | Convergent: 2 (+) 3 (-)  Discriminant: 2 (-) | 2 (+)  5 (-) |
| PMAP (McCallum & Piper, 1992) | English | 109 | Doubtful *n* = 1 | Convergent: 1 (+) | 1 (+) |
| PMAP (Smith et al. 2009) | Dutch | 100 | Very good *n* = 2 | Convergent: 1 (-)  Discriminant: 1 (-) | 2 (-) |
| Psychological construing and defence understanding (Dollinger et al. 1982) | English | 70 | Adequate *n* = 2  Doubtful *n* = 2  Inadequate *n* = 3 | Convergent: 7 (-) | 7 (-) |
| PMS (Andelkovic, 2020) | Serbian | 166 | Adequate *n* = 1 | Discriminant: 1 (+) | 1 (+) |
| PMS (Beitel & Cecero, 2003) | English | 187 | Very good *n* = 6 | Convergent: 2 (+) 3 (-)  Discriminant: 1 (+) | 3 (+)  3 (-) |
| PMS (Beitel, Ferrer & Cecero, 2004) | English | 200 | Adequate *n* = 1  Doubtful *n* = 2 | Discriminant: 1 (+) 2 (-) | 1 (+)  2 (-) |
| PMS (Beitel, Ferrer & Cecero, 2005) | English | 103 | Very good *n* = 7 | Convergent: 3 (+) 1 (-)  Discriminant: 2 (+) 1 (-) | 5 (+)  2 (-) |
| PMS (Conte et al. 1990) | English | 44 | Doubtful *n* = 3  Inadequate *n* = 3 | Convergent: 1(+) 2 (-)  Discriminant: 1 (+) 2 (-) | 2 (+)  4 (-) |
| PMS (Conte et al. 1995) | English | 46 | Doubtful *n =* 14 | Convergent: 5 (+) 3 (-)  Discriminant: 2 (+) 4 (-) | 7 (+)  7 (-) |
| PMS (Conte et al. 1996) | English | 256 | Inadequate *n* = 7 | Convergent: 3 (-)  Discriminant: 1 (+) 3 (-) | 1 (+)  6 (-) |
| PMS (Hua et al. 2007) | Mandarin | 342 | Doubtful *n* = 2 | Not reported (2) | 2 (?) |
| PMS (Sahin et al. 2015) | Turkish | 418 | Adequate *n* = 2 | Convergent: not reported in English (2) | 2 (?) |
| PMS (Shill & Lumley, 2002) | English | 397 | Adequate *n* = 1 | Discriminant: 1 (+) | 1 (+) |
| PMS (Takagishi et al. 2014) | Japanese | 606 | Adequate *n* = 1 | Discriminant: 1 (-) | 1 (-) |
| TAT method (Wolitzky & Reuben, 1974) | English | 14 | Inadequate *n* = 2 | Convergent: 2 (+) | 2 (+) |
